# Educating Midwives as Abortion Providers: Implementing Innovative Models for Standardized Training in Abortion Care

**DOI:** 10.1111/jmwh.70043

**Published:** 2025-12-13

**Authors:** Nicole Quinones, Samantha Auerbach, Nikki Lanshaw, Nikia Grayson, Julie Blumenfeld, Becca Neuwirth, Pamela Pearson, Ellen Chaney Solis, Robyn Nisi, Kylea L. Liese

**Affiliations:** ^1^ Division of Health Policy and Management, School of Public Health University of Minnesota and Division of Complex Family Planning, Department of Obstetrics, Gynecology, and Women's Health, University of Minnesota Medical School Minneapolis Minnesota; ^2^ School of Nursing, University of California San Francisco San Francisco California; ^3^ CHOICES Center for Reproductive Health, Memphis, Tennessee Carbondale Illinois; ^4^ School of Nursing Rutgers, The State University of New Jersey New Brunswick New Jersey; ^5^ College of Nursing University of Illinois Chicago Chicago Illinois; ^6^ School of Nursing University of Washington Seattle Washington

**Keywords:** abortion, advanced practice clinicians, midwifery education, reproductive justice, sexual and reproductive health

## Abstract

The *Dobbs v Jackson Women's Health Organization* US Supreme Court decision significantly limited patients' access to abortion services, and the providers who can legally deliver this care. Currently 22 states license providers other than physicians to provide medication or procedural abortion. However, most midwives and advanced practice clinicians (APCs) do not receive abortion training in their educational programs despite endorsement from professional and health organizations both nationally and abroad. In response to the growing abortion access crisis, 4 accredited midwifery programs incorporated innovative abortion training into their curricula to integrate abortion into the full scope of midwifery practice. This article provides a framework for midwifery and APC programs to implement standardized education and training in abortion services that are trauma informed, culturally sensitive, and patient centered. We outline each program, implementation barriers and facilitators, and conclude with a discussion of mechanisms for overcoming barriers and recommendations for standardization across programs.

## INTRODUCTION

Abortion care has been greatly restricted or banned in 41 states, leading to a crisis of abortion access.^1^ An estimated 1 in 5 patients seeking abortion care are forced to delay care due to travel, often to other states. In addition to increasing patient distress,^2^, ^3^ these delays result in people presenting for abortion at later gestational ages, increasing clinical risk, and health care costs.^4^, ^5^, ^6^ The lack of access to abortion care and providers must be addressed.

**Figure 1:**
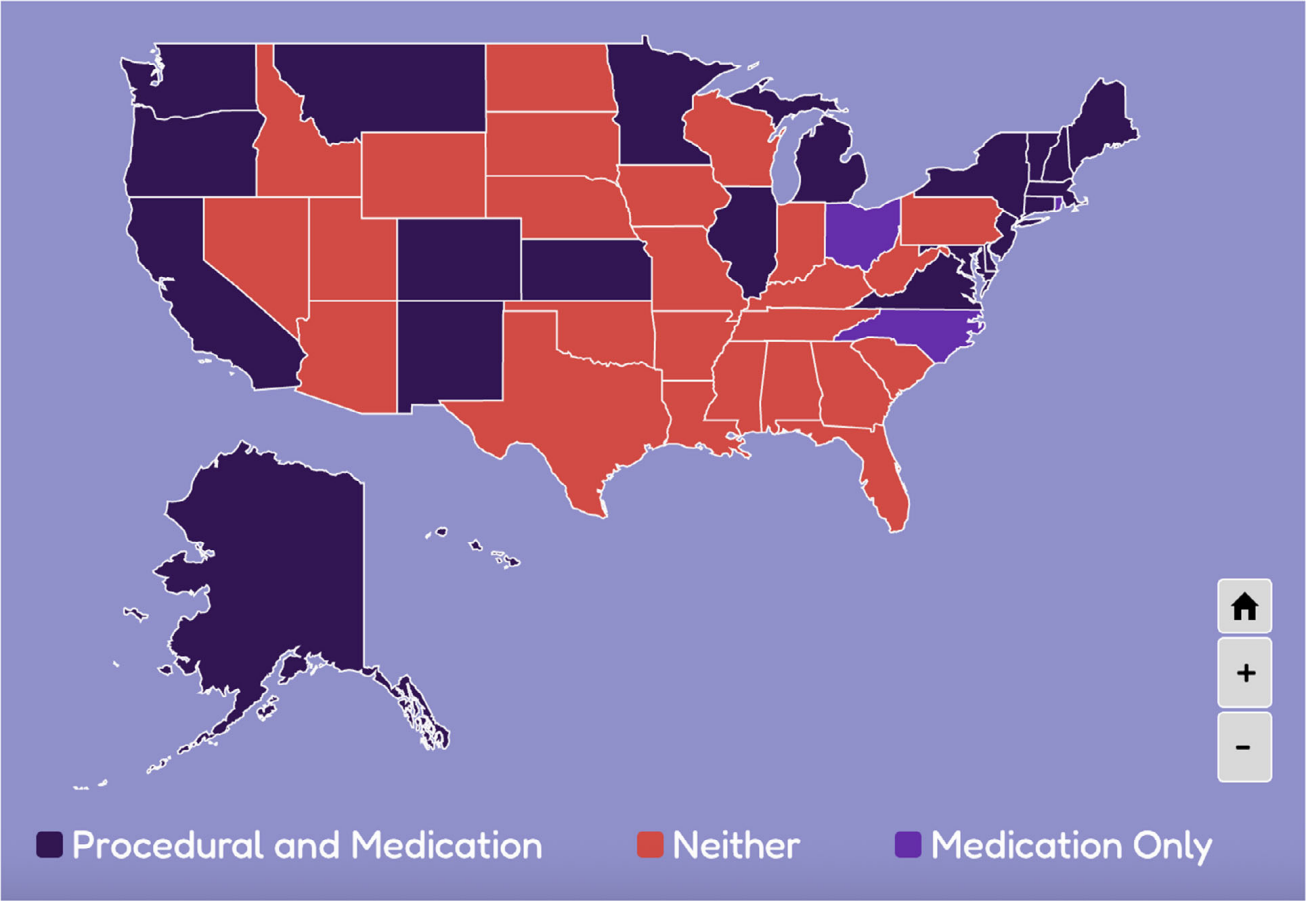
Locations in the United States Where Advanced Practice Clinicians Can Provide Different Types of Abortions Source: Jenkins et al, 2025.^14^

Long recognized^7^ for providing high‐quality, cost‐effective reproductive care, particularly in clinically underserved settings,^8^ advanced practice clinicians (APCs), including certified nurse‐midwives/certified midwives (CNM/CMs), advanced practice registered nurses (APRNs), and nurse practitioners (NPs), are well‐positioned to help alleviate the abortion access crisis. The safety and quality of abortion provision by APCs is well‐established in the literature.^9^, ^10^, ^11^ Inclusion of abortion within APC scope of practice has been endorsed by the World Health Organization^11^; the National Academy of Sciences, Engineering, and Medicine^10^; the American College of Nurse‐Midwives^12^; and the American College of Obstetricians and Gynecologists,^13^ among others.^14^ APCs are legally licensed to provide procedural and medication abortion within 22 states (Figure 1).^15^, ^16^ However, accredited education programs for midwives and advance practice nurses provide little abortion‐related content or clinical opportunities.

  

Continuing education (CE) is available for this article. To obtain CE online, please visit http://www.jmwhce.org. A CE form that includes the test questions is available in the print edition of this issue.

Little research documents the contents of abortion curricula in accredited graduate midwifery programs. A 2006 study noted that only 21% of accredited midwifery graduate programs included clinical instruction of procedural abortion, manual vacuum aspiration, or medication abortion.^17^ Reported barriers to abortion training included lack of curriculum priority (33%), lack of clinical sites (17%), unqualified faculty (33%), and restricted scope of practice due to state law (50%).^17^ Although these findings are almost 20 years old, similar barriers persist highlighting an opportunity for clarification on professional and legal scope of practice^14^ and engagement around the role of APCs in abortion care within educational programs.^18^

In response to the growing abortion access crisis and to ensure midwives and APCs can use their full scope of practice, innovative abortion training programs have been developed within US midwifery programs accredited by the Accreditation Commission for Midwifery Education. This article describes abortion training programs at 4 universities: University of Washington (UW), Rutgers University, University of Illinois Chicago (UIC), and University of California San Francisco (UCSF) (Table 1). Only 1 of the 4 programs is located within a state that licenses CMs (New Jersey). The other 3 states (California, Illinois, Washington) at the current time license only CNMs.^19^

**QUICK POINTS**

✦ Expanding abortion training to midwives and other advanced practice clinicians (APCs) will greatly increase access to services and is endorsed by most major professional organizations.
✦ One major barrier to abortion access is the availability of clinical training sites and preceptors with the capacity and willingness to offer abortion training to APCs, certified nurse‐midwives, and certified midwives.
✦ Advocacy and mobilization of state legislatures are key to the development of training programs, especially in states that maintain total abortion bans, gestational age limitations, and physician‐only laws.

**Table 1:** Description of 4 Midwifery Programs

Program	GCSRH	RADIANT Fellowship	RTEI	Abortion in Primary Care
University and location	University of Washington School of Nursing Nurse‐Midwifery Education Program (Seattle, WA/online, asynchronous)	University of Illinois Chicago College of Nursing Nurse‐Midwifery Program (Chicago, IL)	Rutgers University School of Nursing, Nurse‐Midwifery Program (Newark, NJ)	University of California San Francisco School of Nursing (San Francisco, CA)
State abortion restrictions	Abortions are banned after fetal viability (24‐26 wk), as determined by a health care provider. After viability, abortion is permitted only if the pregnancy endangers life or health.	The Reproductive Health Act of 2019 established a fundamental right to make individual decisions about reproductive health, without government interference. Abortion is legal after viability if it is in the best interest of the mother's mental or physical well‐being.	No major restrictions	Banned after viability (24‐26 wk)
State licensure	Medication and procedural abortion are in the scope of care for licensed APRNs, CNMs, physicians (MD or DO) PAs and pharmacists may only provide medication abortion.	Medication and procedural abortion are in the scope of care for licensed physicians, PAs, and APRN (including CNMs).	CNMs/CMs, APNs, and PAs may provide medication abortion and perform aspiration abortion up to 14 weeks’ gestation.	CNMs and NPs are approved for first trimester aspiration abortion procedures for confirmed intrauterine pregnancy up to 14 weeks’ gestation. NPs require collaborative agreement with MD for at least first 3 y of practice but may provide abortions without supervision otherwise.
Program inception date	September 2024	July 2024	Spring 2023 Midwifery courses began May 2025	Fall 2024
Type	Certificate	Specialty content and elective	Elective and Continuing Education	Elective
Students	APRN, NP, CNM	FNP, PNP, WHNP, CNM, AGPCNP	APRN, CNM	FNP, PNP, CNM, AGPCNP
Curricular content	Clinical training in medication and procedural abortion. Antiracism; reproductive justice; ethical, legal, and cultural dimensions of abortion care	TEACH; complication management, ultrasound, patient‐centered care and counseling, Reproductive justice, practice integration	TEACH, reproductive autonomy, health equity	TEACH curriculum, Reproductive Justice, advanced provision, self‐ managed abortion
Program information	https://nursing.uw.edu/academics/grad‐certificates/sexual‐reproductive‐health/	radiant.uic.edu	Nurse‐midwifery program websites; Rutgers University Center for Professional Development (https://nursing.rutgers.edu/ceiq/professional‐development/)	https://nursing.ucsf.edu/

Abbreviations: AGPCNP, adult gerontological primary care nurse practitioner; APRN, advanced practice registered nurse; CM, certified midwife CNM, certified nurse‐midwife; DO, doctor of osteopathy; FNP, family nurse practitioner; GCSRH, Graduate Certificate in Sexual and Reproductive Health; MD, doctor of medicine; NP, nurse practitioner; PA, physician's assistant/associate; PNP, pediatric nurse practitioner; RADIANT, Reproductive Advocacy and Diversity in Advanced Nursing Training; RTEI, Reproductive Training and Education Initiative; TEACH, Training in Early Abortion for Comprehensive Healthcare; WHNP, women's health nurse practitioner.

As faculty and representatives of these abortion training programs, we outline our respective programs, implementation barriers and facilitators, and conclude with a discussion of mechanisms for overcoming barriers and recommendations for standardization of abortion training for midwives and APCs.

### UW School of Nursing Nurse‐Midwifery Education Program

The UW School of Nursing offers abortion training through a Graduate Certificate in Sexual and Reproductive Health (GCSRH). This program is designed for APRNs and students enrolled in NP or nurse‐midwife programs and provides comprehensive training in advanced sexual and reproductive health, including abortion. The graduate certificate was developed by faculty in the nurse‐midwifery program who identified gaps in APRN education and the ability to provide sexual and reproductive health care. Once conceived, approval of the program required support from UW School of Nursing leadership, faculty, the graduate curriculum committee, and the finance department. After collaborating with these stakeholders, the program began Autumn Quarter 2024. The first cohort of GCSRH students began coursework then and started their clinical experiences in Autumn Quarter 2025.

The GCSRH curriculum is structured around principles of antiracism and reproductive justice, ensuring that students gain clinical expertise and also understand the political, societal, and structural inequities affecting patients’ access to care. The curriculum includes a combination of didactic coursework and clinical training, emphasizing patient‐centered, trauma‐informed, and culturally sensitive care. The program aims to normalize these services by integrating abortion care into a broader framework of sexual and reproductive health care.

The GCSRH faced significant barriers to providing abortion care education, even in a state without restrictive abortion laws. One major barrier was the availability of clinical training sites with the capacity and willingness of providers to offer abortion rotations to NPs and CNMs. Another critical barrier was the training cost, which can be prohibitive for many students. Tuition fees for graduate‐level certificate programs, travel costs to clinical sites, and licensing or certification examination fees may deter students from enrolling in these programs.

To successfully implement the abortion care education program at UW, it has been critical for the program to have strong institutional leadership, faculty commitment to reproductive justice, and collaboration with community partners. Program leadership has continued to foster relationships with clinics that can provide students real‐world experiences that enrich their learning. Online and hybrid teaching models increased accessibility for students across diverse geographic regions. Embedding the curriculum within a broader context of sexual and reproductive health care has allowed students to understand abortion care as a vital component of overall health and well‐being. By addressing clinical competencies and the ethical, legal, and cultural dimensions of care, the program has ensured that students are well‐prepared to meet the needs of diverse patient populations.

### Rutgers University Midwifery Program

In 2021, New Jersey repealed physician‐only requirements for procedural abortion to increase access to abortion care.^20^, ^21^ In 2023 the New Jersey Division of Consumer Affairs awarded Rutgers University School of Nursing funding to develop the Reproductive Training and Education Initiative (RTEI) to provide abortion and reproductive health education and training to advanced practice students and licensed providers in New Jersey. This included updating abortion‐related content within all APC curricula at Rutgers offering day‐long abortion care workshops for licensed APCs, which incorporated both didactic and simulation content, as well as opportunities for clinical placement.

The New Jersey Board of Medical Examiners (BME) Midwifery Liaison Committee subsequently created rules specifying the education and training required for New Jersey licensed CNMs/CMs to provide procedural abortion.^20^ In response, RTEI developed a course that exists as continuing education for licensed midwives through the Rutgers School of Nursing Center for Professional Development and an elective within the Rutgers Midwifery Program curriculum. This course provides CNMs/CMs and midwifery students the opportunity develop necessary skills and competencies to provide procedural abortion care in accordance with BME rules. Emphasizing reproductive autonomy and health equity, the didactic coursework, clinical education, and training are based on the Bixby Center for Global Reproductive Health's interactive Training in Early Abortion for Comprehensive Healthcare (TEACH) curriculum (Table 2). It is a primarily asynchronous, 11‐week summer course that includes (1) 9 units with recorded presentations, facilitated by midwives, APRNs, and physicians, corresponding to the chapters in the TEACH manual; (2) 3 in‐person skills labs; and (3) 50 hours of hands‐on clinical experience. The midwifery graduate students are required to complete additional assignments. The grant provides annual funding for 3 years for 5 learners to enroll in each of the courses and financial support for preceptors. Upon completion of these courses, learners receive documentation that they can present to the BME.

**Table 2:** Training in Early Abortion for Comprehensive Healthcare Abortion Training Curriculum Outline (Eighth Edition)^a^

Chapter Title	Chapter Sections
Abortion in perspective	Reproductive health through a justice lens
	Global abortion facts at a glance
	United States abortion facts at a glance
	Overview of US abortion law
	United States law and policy highlights
	Adoption facts at a glance
	Program overview and core competencies
Counseling	Addressing systemic and personal bias
	Pregnancy options counseling techniques
	Responding to challenging questions
	Abortion options
	Self‐managed abortion
	Confidentiality
	Informed consent
	Making referrals
	Addressing diverse needs
Preabortion evaluation	Pregnancy confirmation and dating
	Health evaluation prior to uterine aspiration
	Ultrasound overview: methods, tips, and images
	Ultrasound findings with abnormal pregnancies
	Pregnancy of unknown location evaluation
Medication abortion	Comparison of medication abortion regimens
	Mifepristone/misoprostol abortion <14 wk: Step by step
	Ultrasound as needed with medication abortion
	Self‐managed medication abortion, menstrual regulation, and advanced provision
	Managing complications associated with medication abortion
Pain management and managing emergencies	Preprocedure medications
	Pain management
	Pain management considerations for people who use drugs
	Basic medication options
	Managing emergencies
Uterine aspiration	Trauma‐responsive care during procedures
	Quick Guide: Communication during the procedure
	“No‐Touch” technique
	Steps for uterine aspiration
	Using MVA and EVA equipment
	Abortion aftercare
	Abortion procedure aftercare
	Managing complications
Contraception	Ensuring equitable, accessible, and high‐quality contraception
	Contraceptive counseling
	Evidence‐based contraceptive guidance
	Method specific information and considerations
	Addressing diverse needs
	Medical eligibility for initiating contraception
Early pregnancy loss	EPL
	Counseling tips for early pregnancy loss
	EPL diagnostic and clinical considerations
	Comparing management options for EPL
	EPL options counseling
Becoming a provider	Building and maintaining your skills
	Leadership, advocacy, and policy
	Finding practice opportunities
	Personal security
	Organizational and patient resources

Abbreviations: EPL, early pregnancy loss; EVA, electric vacuum aspiration; MVA, manual vacuum aspiration.

a Additional optional chapters include Practice Integration, Becoming a Trainer, Incremental Expansion, and Evaluation. See https://teachtraining.org/abortioncurriculum.

Source: Fleming et al, 2025.^22^

Various factors supported or impeded the development and implementation of these courses. The current New Jersey state administration's support for abortion rights led to state policy changes and subsequent funding that enabled the development of these courses. Additionally, the strong support from both Rutgers’ Medical School physician colleagues and community physicians engaged in abortion care led to interprofessional collaborations to develop and implement the diverse components of RTEI. Limited availability of clinical sites and preceptors was a primary challenge for the development, implementation, and growth of this course.

### UIC College of Nursing Nurse‐Midwifery Program

Illinois experienced the largest increase in patients traveling from out of state for abortion care since the 2022 *Dobbs* decision overturned *Roe v Wade*.^23^ In January 2023, Governor Pritzker signed HB 4664 affirming that medical and procedural abortion are part of the scope of care for CNMs and APRNs, an important step to expanding abortion access in Illinois. Subsequently, UIC College of Nursing was awarded funding from the Illinois Department of Public Health to establish the Reproductive Advocacy and Diversity in Advanced Nursing Training (RADIANT) Fellowship. The RADIANT program developed a sustainable model for advancing abortion care by combining curricular changes for APRN and CNM student specialties with targeted abortion training of licensed providers.

As licensed providers, RADIANT fellows are prepared to provide abortion using a curriculum that includes an adaptation of the TEACH curriculum with content specific to providing abortions in Illinois. Upon completing online abortion and ultrasound modules, fellows participate in an intensive 2‐day in‐person simulation. Simulations include interactive and hands‐on ultrasound training, manual vacuum aspiration with dragon fruit, complication management simulations, and standardized patients to practice trauma‐informed, gender‐affirming abortion care and counseling. The fellowship also includes quarterly seminars on practice integration and mentorship with experienced providers. Upon completion of the program, fellows receive a completion certificate for documentation.

UIC faculty implemented a 3‐step approach for training students in advanced prctice nursing and nurse‐midwife programs to safely integrate abortion care into their practices after graduation. First, all specialties (family NP, pediatric NP, CNM, women's health NP, and adult gerontological primary care NP) implemented standardized didactic abortion care content led by RADIANT faculty. The curriculum includes full‐day simulations for NP and CNM students each semester with topics such as options counseling, medication abortion, limited dating ultrasound, a procedural abortion workshop, and advanced long‐acting reversible contraception placements. All students participate in values clarification workshops prior to simulations. These training opportunities, open to all doctor of nursing practice (DNP) students wanting to learn more about abortion management, will be integrated into an elective course on Advanced Topics in Reproductive and Sexual Health and include didactic and clinical experiences in abortion management. The elective course sets the foundation for a certificate program for students or licensed providers seeking to advance their training. Modeled after the UW, UIC's certificate program will also include content on the history of abortion access, reproductive justice principles, advocacy, and strategies for integrating these concepts into clinical practice.

Implementing this program included several barriers. Faculty had difficulty accessing adequate clinical training to meet hospital requirements for privileges, complicating their ability to provide services and train students and fellows. Most abortion training opportunities available at UIC are reserved for medical students, fellows, and obstetrics and gynecology residents. Even when a half‐day abortion clinic to train CNMs was created, the clinic was unable to offer moderate sedation, which restricted case volume and faculty's ability to achieve competency or privileging. Few high‐volume clinics outside of UIC were willing to open additional training spots, and existing institutional training agreements did not include APRN or CNM faculty, which required new agreements and independent malpractice insurance.

Despite these barriers, several factors have facilitated the successes of the innovative abortion training model at UIC, including grant funding from the state, administrative support, and an interprofessional team‐based approach. We have also established relationships with independent clinics to build training opportunities for faculty and students. Positive engagement across specialty program directors has been critically supportive to building curricula that meets the needs of all students across a variety of practice settings.

### UCSF School of Nursing

The UCSF abortion training opportunities were funded by the California Department of Health Care Access and Information, via California's Reproductive Health Service Corps, a consortium of partners to develop and implement evidence‐based abortion care training programs across health professions and increase the number of abortion providers in California. UCSF School of Nursing is responsible for creating and implementing didactic, simulation, and clinical training opportunities for advanced practice nursing students and licensed APRNs.

Using the TEACH curriculum as a framework, faculty designed and integrated abortion electives (didactic, simulation, and clinical) into the UCSF School of Nursing Master's Entry Nurse Practitioner (MEPN) program. Didactic material included integrating abortion services into primary care, including Federally Qualified Health Centers, advance provision medication abortion, and the clinician's role in self‐managed abortion. Abortion‐specific content in the simulation course is being implemented and will provide the opportunity to practice uterine aspiration techniques and ultrasound in early pregnancy and demonstrate patient‐centered trauma‐informed counseling techniques with standardized patients. Clinical placement opportunities will be available for students to gain real‐world clinical experiences with abortion.

The primary barrier for the creation and implementation of this program was the limited time allotted for elective courses. We had intended the abortion content to continue as elective courses; however, the MEPN program transitioned to a post–bachelor of science in nursing (BSN) to DNP program in 2024. The additional coursework required for the new DNP program maxes out students’ schedules and electives were deprioritized. Given these constraints, faculty champions are creatively and flexibly integrating abortion content across required coursework for DNP students. Abortion‐related clinical skills are being integrated into a required, preexisting course, alongside student exposure to other common in‐office procedures, such as intrauterine device placement and abscess incision and drainage. All UCSF APRN students will be exposed to the content, effectively institutionalizing abortion into general primary care content. The availability of grant funding also helped overcome administrative barriers. We were able to *buy out* time from a group of faculty representing all relevant NP specialties within the School of Nursing with experience in abortion provision, curriculum building, and skills laboratory development, which supported program development.

## DISCUSSION

Midwives and APRNs are a critical part of the solution to increase abortion access. Training programs must address barriers to implementing didactic and hands‐on clinical training for abortion. Four innovative midwifery programs at accredited institutions have developed and implemented standardized education and training in abortion services that are trauma informed, culturally sensitive and patient centered. These programs normalize abortion education, training, and provision within sexual and reproductive health care practice by incorporating it into standardized training. Faculty in each institution led processes from inception to implementation of these programs with various factors impeding or facilitating their development and success. Several common themes emerged that facilitate building similar training programs at other institutions. It is important to note that coupling these programs with educational and state policy changes can facilitate the inclusion of abortion in standard APRN and midwifery education.^24^ These recommendations are intended to bridge the clinical skill gap in miscarriage management as well as prepare APRNs for emergency situations in which abortion procedures must be provided.

### Training Opportunities

Abortion exceptionalism, or treating abortion differently and separately from other health care procedures through mechanisms such as overregulation (eg, requiring high quotas to establish competency), is responsible for many of the operational barriers these training programs faced.^25^, ^26^ When evaluating competency, institutions should shift to using standardized competency‐based evaluations, which are currently available and recommended as research best‐evidence.^27^, ^28^ By relying upon quotas, institutions are setting ill‐advised precedents from which other institutions follow, ossifying numbers required for training and privileging, and creating a new set of downstream barriers for providers and students.

The lack of adequate sites for clinical training opportunities, another common barrier, has been an ongoing issue in abortion training for decades.^29^ Quota‐based trainings contribute to a lack of available clinical placement spots, but the spots that do exist often prioritize medical students and residents, limiting APRN and CNM placements. Including abortion as a component of primary health care across APRN professional bodies could generate additional training opportunities and dedicated placements. The Accreditation Council for Graduate Medical Education requires obstetrics and gynecology residents receive routine abortion training, and this had led to the creation of specific programs to facilitate training rotations, such as the Ryan Program^30^ and the Fellowship in Family Planning.^31^ Of note, UCSF reported sufficient training sites and attribute this to using state funds to pay clinics for taking students.

### Interprofessional Collaboration

Collaboration and standardization between accredited programs at different stages of developing their abortion educational programs will generate the highest quality training and providers. The programs described here meet regularly to share didactic and simulation content, do administrative paperwork, and build community. Future plans to support sustainability and expansion for the 4 programs described here include (1) standardizing program evaluation criteria to support continued innovation and research and (2) developing a toolkit that includes curriculum and resources for other institutions to integrate abortion training into their programs.

## CONCLUSION

Expanding the scope of abortion training beyond physician‐only models can significantly enhance health care access and improve outcomes for patients, particularly in rural and underserved communities where barriers to care are greatest. APCs are critical to increasing abortion access. Advance practice nursing and midwifery programs must include didactic and hands‐on clinical training for abortion and address barriers to implementing comprehensive abortion training. Standardizing abortion training curricula requires administrative efforts to secure clinical placements and funding for students. Expansion of APC abortion training can be assisted by learning from the experiences of these 4 examples, particularly by noting barriers to implementation and proactively planning for solutions to help ensure program success. Inclusion of abortion in routine nurse practitioner and midwifery education programs is a critical step in reintegrating abortion into health care and alleviating the abortion access crisis. Innovations in the programs described here provide an important roadmap for other educational programs seeking to build a health care workforce that leverages the full capacity of their knowledge and skills to meet these challenges. Equipping APCs with abortion care skills helps dismantle silos in health care and ensures that more patients receive timely, compassionate, and comprehensive reproductive health services.

Research presented here is funded in part by the Illinois Department of Public Health. The programs we reviewed all received funding to train students and professionals in abortion. The amount and source of funding received varied, allowing different levels of educational infrastructure development, such as ultrasound simulation equipment, training placements, and financial support for students. Supportive nursing leadership combined with funding facilitated building robust, multidisciplinary and sustainable abortion training programs to prepare students to take on the clinical, social, and political barriers to providing abortion care. Recognizing funding and leadership support can vary across institutions, while the need for trained clinicians remains high, the educational content of these innovative programs is largely open and accessible to all. APRN advocacy and mobilization at state legislatures are key to abortion access advocacy. Advocacy is also necessary for the development of training programs and partnerships especially with APRN and midwifery programs in states that maintain total abortion bans, gestational limits and physician‐only laws.

## CONFLICT OF INTEREST

The authors have no conflicts of interest to disclose.
